# PReS-FINAL-2185: Prognostic markers in juvenile vs. adult-onset ankylosing spondylitis

**DOI:** 10.1186/1546-0096-11-S2-O20

**Published:** 2013-12-05

**Authors:** D Jadon, LP Hunt, R Arumugam, AV Ramanan, R Sengupta

**Affiliations:** 1 Rheumatology, Royal National Hospital for Rheumatic Diseases, Bath, UK; 2 Paediatric Rheumatology, University Hospital Bristol NHS Trust, Bristol, UK

## Introduction

Patients experiencing ankylosing spondylitis (AS) symptoms ≤16 years-of-age are classified as juvenile-onset AS (JoAS), whilst those ≥17 years-of-age adult-onset AS (AoAS). Studies from North America, China and Turkey suggest that JoAS and AoAS patients have differing clinical characteristics and functional outcomes; although results have been inconsistent.

## Objectives

This study compared JoAS *vs*. AoAS with respect to clinical, functional and genetic outcomes, and determined which factors were related to prognosis, as defined either by a poor BASFI (≥5) or by a history of AS-related surgery.

## Methods

143 JoAS were compared with 413 AoAS patients attending a secondary care rheumatology hospital.

A diagnosis of AS was made using the 1987 modified New York criteria. The following clinical parameters were recorded: sex; age at symptom onset (JoAS only); age at Rheumatologist-made diagnosis; the most recent BASFI, BASDAI, BASMI; *HLA-B27 *genotype status; the occurrence at any time point of psoriasis, uveitis, enthesitis, inflammatory bowel disease (IBD) or AS-related surgery (hip, shoulder or spinal).

Two group comparisons were made with continuity-corrected Chi-squared, unpaired Student's t-tests and non-parametric Mann-Whitney U-tests. Logistic regression was used to adjust for time since diagnosis.

## Results

At assessment, JoAS cases were slightly younger than AoAS cases (mean age 49.0 *vs*. 51.9 years; mean difference in age 2.9 years, 95% CI 0.3-5.6 years). JoAS cases had a slightly longer mean disease duration since diagnosis than AoAS cases (26.0 years *vs*. 19.3 years).

JoAS cases were more likely to have had AS-related surgery than AoAS (18.9% *vs*. 8.0%, respectively; p < 0.001; or p = 0.017 after adjustment for time from diagnosis), and slightly more had had concurrent IBD (11.2% *vs*. 6.8%; p = 0.13).

No statistically significant difference was found between the two groups in terms of BASFI, ten BASFI domains, BASDAI, BASMI, sex distribution, *HLA-B27 *positivity, psoriasis, enthesitis, or uveitis (all cases or *HLA-B27 *positive cases only).

JoAS cases with psoriasis were more likely to have a poor BASFI (≥5.0) than those without psoriasis (55% *vs*. 25%; p = 0.016), and were also more likely to have had AS-related surgery than those without psoriasis (43% *vs*. 15%; p = 0.006).

JoAS cases with a poorer BASFI showed a trend for symptom onset at a younger age than those with a better BASFI (<5.0) (mean age 12.5 *vs*. 13.4; p = 0.08). Similarly, JoAS cases having had AS-related surgery showed a trend for symptom onset at a younger age than those without surgery (mean age 12.5 *vs*. 13.3; trend p = 0.18).

## Conclusion

This study is the first to investigate a Northern-European population of Rheumatologist-diagnosed JoAS patients, and is the largest sample of prospectively-collected JoAS data published. JoAS and AoAS patients differed in terms of proceeding to AS-related surgery, and occurrence of IBD. In JoAS, younger age at symptom onset and occurrence of psoriasis, related to poorer prognosis. Delayed diagnosis of JoAS didn't correlate with prognosis.

## Disclosure of interest

None declared.

